# Interactive Effects of Arbuscular Mycorrhizal Fungi and Copper Stress on Flowering Phenology and Reproduction of *Elsholtzia splendens*

**DOI:** 10.1371/journal.pone.0145793

**Published:** 2015-12-28

**Authors:** Zexin Jin, Junmin Li, Yueling Li

**Affiliations:** 1 Zhejiang Provincial Key Laboratory of Plant Evolutionary and Conservation, Taizhou, China; 2 Institute of Ecology, Taizhou University, Taizhou, China; Swiss Federal Research Institute WSL, SWITZERLAND

## Abstract

Plant responses to heavy metal contamination may depend on the presence of arbuscular mycorrhizal fungi (AMF). *Elsholtzia splendens* is an indicator species for the presence of copper (Cu) mines because both its flowering phenology and reproduction are tolerant to heavy metals. To test whether effects of Cu on the flowering phenology and reproduction of *E*. *splendens* depend on the presence of AMF, we conducted a factorial experiment with two Cu treatments (with or without Cu addition) crossed with two AMF treatments (with or without AMF inoculation). Without AMF, Cu addition significantly delayed the onset dates, ending dates and peak dates of flowering and decreased flowering duration. However, AMF inoculation reversed the effects of Cu stress, with recovered flowering onset and ending dates and increased the flowering duration. Cu addition significantly decreased inflorescence width and number, inflorescence biomass, vegetative biomass and total seed number, but significantly increased 1000-seed weight. AMF inoculation significantly increased vegetative biomass. Two-way ANOVA results showed that the interactive effects between Cu addition and AMF inoculation were significant on the inflorescence number, vegetative biomass and total seed number. These results indicate that AMF can alleviate the Cu stress on the flowering phenology and reproduction of *E*. *splendens*.

## Introduction

Copper (Cu) is a common trace element that occurs naturally in soil, and plays an essential role in plant growth [1]. Cu is important for the synthesis of enzymes and proteins that are used by plants for various metabolic processes [1]. However, high Cu concentration is highly phytotoxic and can inhibit the photosynthetic and metabolic pathways in plants, thus resulting in decreased biomass and delayed flowering and fruiting [2–4].

Arbuscular mycorrhizal fungi (AMF) are commonly associated with roots of plants, forming symbionts [5]. It is well documented that AMF can help plants adapt to heavy metal stress such as Cu [5–7]. Recently, AMF was used as a Cu toxicity alleviator to enhance metal tolerance in plants, thereby ensuring their survival in Cu-contaminated soils [7–9]. The alleviating effects of AMF are reported to reduce the metal uptake, restricte the translocation of metals to the shoots, improve the soil structure and the nutritional status of plants, and biosorpt the metal sequestration onto the cell wall [8–12]. To date, however, few studies have focused on the interactive effect of Cu and AMF on plant flowering phenology and reproduction.

Flowering phenology is critical to the survival and reproduction of plants [13–15], and reproductive output is important for the long-term persistence of the plant species [16]. Both flowering phenology and reproduction are very sensitive to heavy metals [17]. For instance, soil contamination by heavy metals delayed flowering phenology of *Hieracium pilosella* [17,18] and reduced sexual reproduction of plants [19]. On the other hand, AMF can increase reproductive output and also change flowering phenology [19,20]. We thus hypothesized that AMF will attenuate the negative effects of Cu stress on flowering phenology and reproduction of plants.

*Elsholtzia splendens* is an annual herb that belongs to the Labiatae family. *E*. *splendens* is an indicator of Cu mines and is widely distributed on Cu mining wastes and Cu-contaminated soils along the middle and the lower reaches of the Yangtze River, China [21,22]. This species is Cu tolerant and thus has been used as an Cu-accumulation plant [23]. Previous studies have focused on biochemical and physiological responses of *E*. *splendens* to Cu stress, the ability of *E*. *splendens* to accumulate metal, and the chemical forms of Cu that exist in *E*. *splendens* [23,24]. Roots of *E*. *splenden*s were found to form symbiont with AMF [25], and AMF plays an important role in the absorption and accumulation of heavy metals in *E*. *splendens* [26]. Here, we conducted a pot experiment to test the potential interactive effects of Cu and AMF on flowering phenology and reproductive allocation of *E*. *splendens*. Specially, we aimed to answer the question of how Cu and AMF interact to affect the flowering phenology and reproductive allocation of E. spelendens. These results could serve as a basic reference for the selection of Cu-tolerant or Cu-resistant plants used for phytoremediation of Cu-contaminated soils.

## Materials and Methods

### Experimental design

Plants were propagated in potting soil that was a mixture of peat soil, sand and vermiculite, at a volume ratio of 6:3:1. The soil mixture was autoclaved at 121°C for 2 h to eliminate native AMF propagules and other soil biota [27]. The autoclaved soil mixture had a pH of 5.73 ± 0.04, with an organic matter content of 20.16 ± 0.26 g·kg^-1^, total nitrogen content of 14.61 ± 0.53 mg·kg^-1^, available phosphorus content of 17.86 ± 0.49 mg·kg^-1^, and available potassium content of 56.67 ± 0.16 mg·kg^-1^ soil.

On December 20, 2012, *E*. *splendens* seeds were collected from plants grown in clean soil (without Cu contamination) on an abandoned field in Tainan village (31°30.632’N, 114°32.620’E; altitude 118 m), Hong’an County, Hubei Province, China, and stored at low humidity at room temperature. No specific permissions were required for collecting the seeds.

On December 21, 2012, soil from a depth of 0 to 20 cm was collected from Cu mine tailings located on Chimashan Mountain (29°59.776’N, 115°05.856’E; altitude 138 m), Yangxin County, Hubei Province, China. The soil type was sandy, and the soil texture was sandy clay. The vegetation on the tailings was dominated by *E*. *Splendens* with some pioneer plant species, including *Cynodon dactylon*, *Xanthium sibiricum*, *Artemisia capillaries*, *Silene fortunei* and *Commelina communis*. The soil was air-dried and passed through a 2-mm sieve to remove plant residues, large stones and soil fauna, and was then stored at -20°C for further use as the source of soil microbes.

On May 1, 2013, pots (19 cm in inner diameter and 15 cm deep) were sterilized using 75% ethanol and then filled with 1.7 kg sterilized soil mixture. Pots were placed on the ground in a greenhouse under natural light and ambient temperature. The pot locations within the greenhouse were randomized. Four treatments, coded as -Cu-AMF (no AMF and no Cu), +Cu (Cu addition), +AMF (AMF inoculation) and +Cu+AMF (Cu addition and AMF inoculation), were included in the experiment. A total of 60 pots, with 15 replicates for each treatment, were used. On May 5, 2013, 50 ml liquid with a concentration of 34 mg/ml CuSO_4_·5H_2_O was applied to each pot in the +Cu and +Cu+AMF treatments. On May 6, 2013, the soil collected from Cu mine tailings was incubated for 48 hours at room temperature. A total of 85 g soil was extracted with 50 ml Milli-Q water (Millipore Cooperation, Bedford, MA, USA); this extract was then applied to each pot in the -Cu+AMF and +Cu+AMF treatments. For the -Cu-AMF treatment and the +Cu-AMF treatment, 50 ml extraction water was filtered through 2 mm, 1 mm, 0.5 mm, 0.1 mm, 0.075 mm and 11 μm Whatman filter paper, and the filtrate was applied to the appropriate pots.

On May 5, 2013, *E*. *splendens* seeds were surface-sterilized with 0.5% NaClO, washed several times with sterilized distilled water and then sowed in trays containing the autoclaved soil mixture; seeds maintained in a greenhouse at Taizhou University (121°17’E, 28°87’N), Linhai City, Zhejiang Province, China for germination. On June 5, 2013, 12-cm-tall seedlings were transplanted into experimental pots with one seedling per pot. Pots were watered with tap water as necessary, and their weight was monitored to ensure that the soil moisture content was consistent.

### Measurement

*E*. *splendens* has a compound spike verticillaster inflorescence. The phenology of individual *E*. *splendens* was monitored by determining the inflorescence level every two days from October 10 to November 10, 2013 [28]. The onset of flowering for each individual was recorded as the date on which each spikelet had at least one open floret. The date when 25% individuals were flowering were recorded as the onset date of the treatment. The peak flowering date was recorded as the date on which 50% of the spikelets had more than one opened floret. The end of flowering was recorded as the date on which 95% of the spikelets had more than one open floret. The duration was the difference between the first date on which an open floret was observed and the date on which the last floret opened across all spikelets.

On the peak flowering date, the inflorescence size, including length and width, was measured using Vernier calipers with 0.01 mm precision. When all the seeds matured, the plants were harvested. Plants were separated into vegetative structures, seeds and inflorescences. The numbers of flowers and seeds were recorded, and the weight of 1000 seeds was calculated. The biomass of vegetative structures, seeds and inflorescences were measured.

Fine roots were sampled and the success of AMF inoculation was verified by the mycorrhizal colonization rate. Fine roots were cut into 1-cm-long segments and fixed using formalin acetic alcohol fixation solution. Root samples were cleaned with 10% KOH solution at 90°C for 40 min, acidified in 2% HCl for 5 min, stained with 0.01% acid fuchsin [29], and observed by microscopy. A root segment was considered to be mycorrhizal when arbuscules, vesicles, or intercellular hyphal coils could be clearly identified. The mycorrhizal colonization rate was calculated using the following formula: AMF colonization rate (%) = 100 × root length infected/root length observed [30].

### Statistical analysis

Data are presented as the mean ± 1 standard deviation. A two-way analysis of variance (ANOVA) was used to test the effect of AMF (inoculated vs. uninoculated) and Cu (added vs. not added) on the growth of *E*. *splendens*, with Cu and AMF as fixed factors. A least significant difference (LSD) test was employed to compare the treatment effects (-Cu-AMF, +Cu-AMF, -Cu+AMF, +Cu+AMF) at the 0.05 level of significance. All analyses were performed using the SPSS 17.0 software package for Windows. All figures were created in SigmaPlot 11.0.

## Results

### AMF colonization

**Fig 1:**
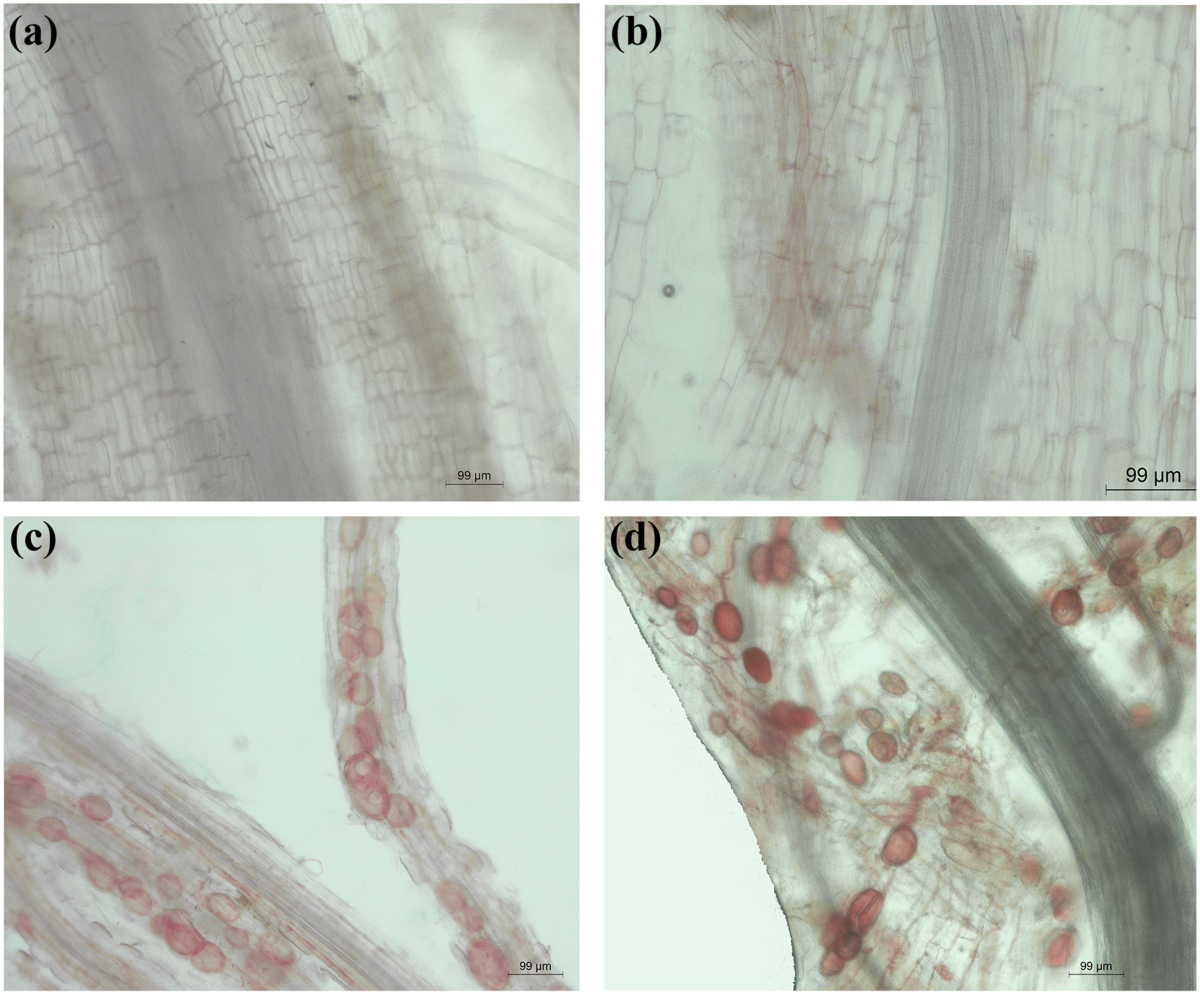
Microscopy of the fine roots of *E*. *splendens* under the four treatments. (a) -Cu-AMF treatment (no AMF and no Cu), (b) +Cu-AMF treatment (Cu addition), (c) -Cu+AMF treatment (AMF inoculation), (d) +Cu+AMF treatment (Cu addition and AMF inoculation).

The AMF colonization rate for the -Cu+AMF and +Cu+AMF treatments was 42.5% and 52.8%, respectively, and the rate for the -Cu-AMF and +Cu-AMF treatments was 0 (Fig 1). These findings indicated that the inoculation with AMF was successful for both +AMF and +Cu+AMF treatments.

### Effects of Cu and AMF on flowering phenology

Cu addition delayed the onset and end dates of flowering by up to 18 and 11 days, respectively, whereas AMF inoculation advanced the onset and end dates, regardless of whether Cu was added or not (Table 1). Cu addition delayed the peak flowering date, whereas AMF inoculation alone had no effect on the peak flowering date but advanced that of *E*. *splendens* under Cu stress (Table 1). Cu addition decreased the duration of flowering by 14.4 days, whereas AMF inoculation increased flowering by 1.4 days in the absence of Cu and by 6.5 days in the presence of Cu (Table 1).

**Table 1:** Phenology data for *E. splendens* in the four experimental treatments.

	-Cu-AMF	+Cu-AMF	-Cu+AMF	+Cu+AMF
Onset	Oct. 6	Oct. 24	Oct. 4	Oct. 20
Duration	36.6±2.0a	22.2±2.6b	38.0±2.5c	28.7±2.4d
Peak flowering date	Oct. 23±2.3b	Nov. 8±2.7a	Oct. 23±1.4b	Nov. 6±1.7a
End date	Nov.15	Nov. 26	Nov.14	Nov. 22
Amplitude (flower/plant/day)	3.38±2.75a	4.44±1.97a	3.09±2.27a	4.57±3.67a

Note: Values are given as the mean ± standard deviation (n = 15). Different small letters in the same line indicate the significant difference at *P*< 0.05. -Cu-AMF indicates no AMF and no Cu, +Cu-AMF indicates Cu addition, -Cu+AMF indicates AMF inoculation, +Cu+AMF indicates Cu addition and AMF inoculation.

**Fig 2:**
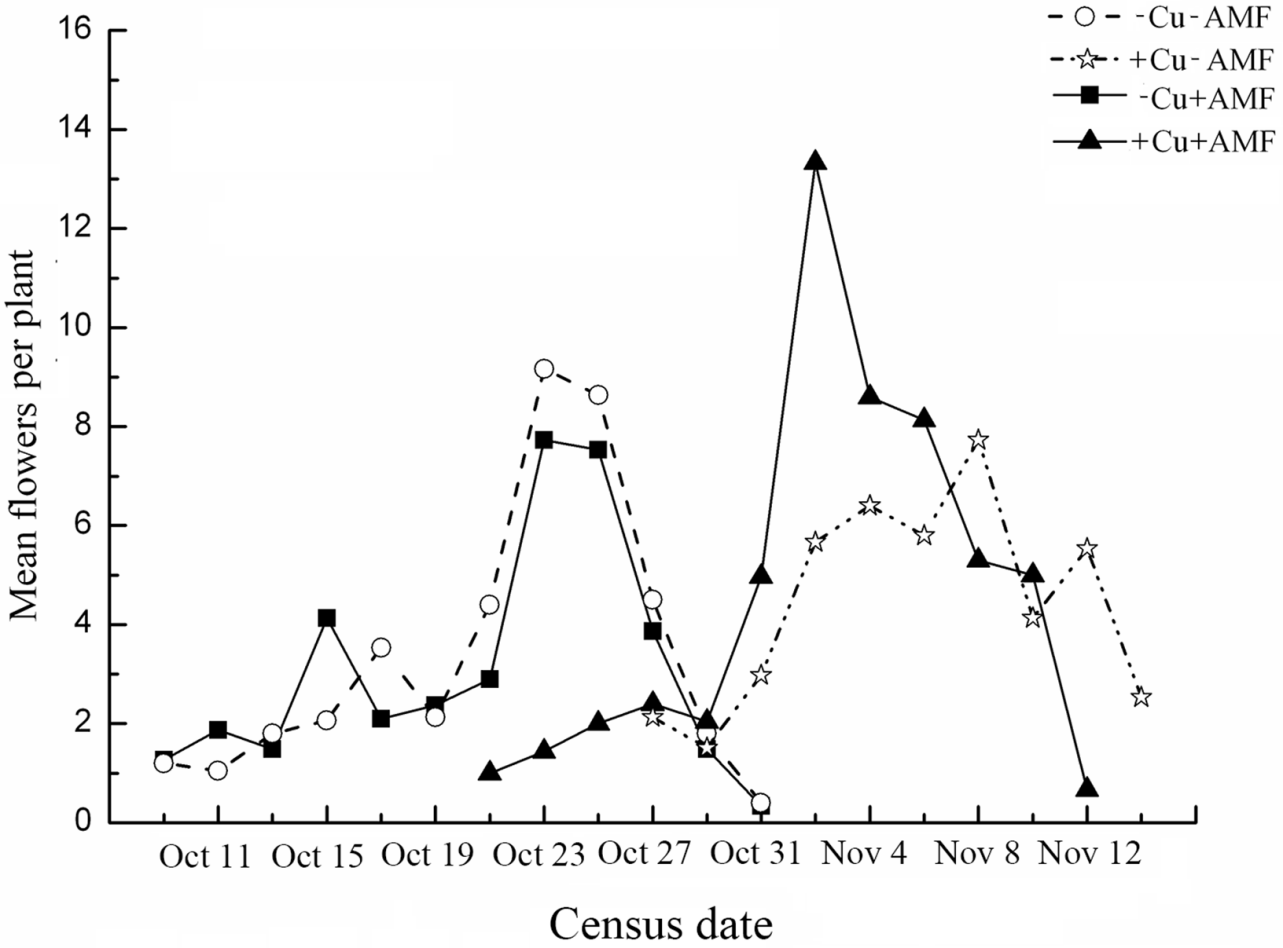
Mean flowering amplitudes for *E*. *splendens* under different treatments. Census intervals were 2 days. Amplitude shown is the mean number of flowers per day. -Cu-AMF indicates no AMF and no Cu, +Cu-AMF indicates Cu addition, -Cu+AMF indicates AMF inoculation, +Cu+AMF indicates Cu addition and AMF inoculation.

The flowering process of *E*. *splendens* in the four treatments showed a single peak process (Fig 2). AMF inoculation decreased the mean flowering amplitude, whereas Cu addition increased the amplitude, regardless of AMF inoculation (Table 1 and Fig 2).

### Effects of Cu and AMF on *E*. *splendens* reproduction

**Fig 3:**
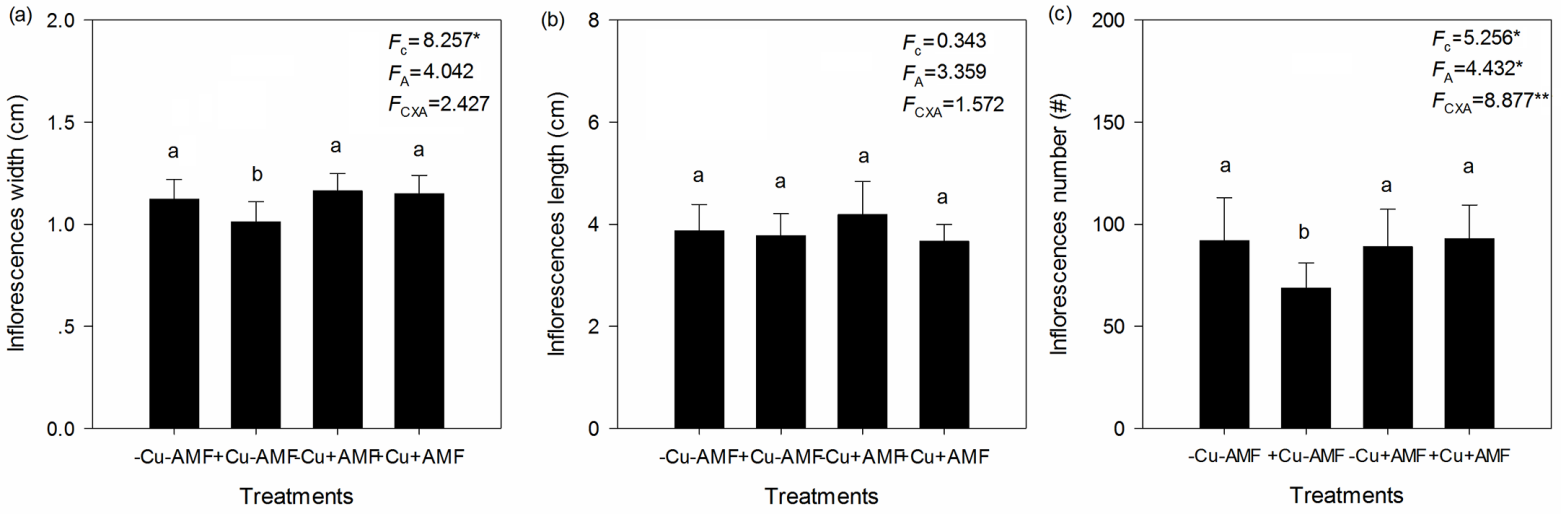
Effects of Cu addition and AMF inoculation on inflorescence length (a), width (b) and number (c). -Cu-AMF indicates no AMF and no Cu, +Cu-AMF indicates Cu addition, -Cu+AMF indicates AMF inoculation, +Cu+AMF indicates Cu addition and AMF inoculation.

Cu addition significantly decreased inflorescence width and number, but had no effect on inflorescence length (Fig 3). AMF inoculation and the interaction between Cu addition and AMF inoculation had no effect on inflorescence length or width, but did significantly affect inflorescence number (Fig 3).

**Fig 4:**
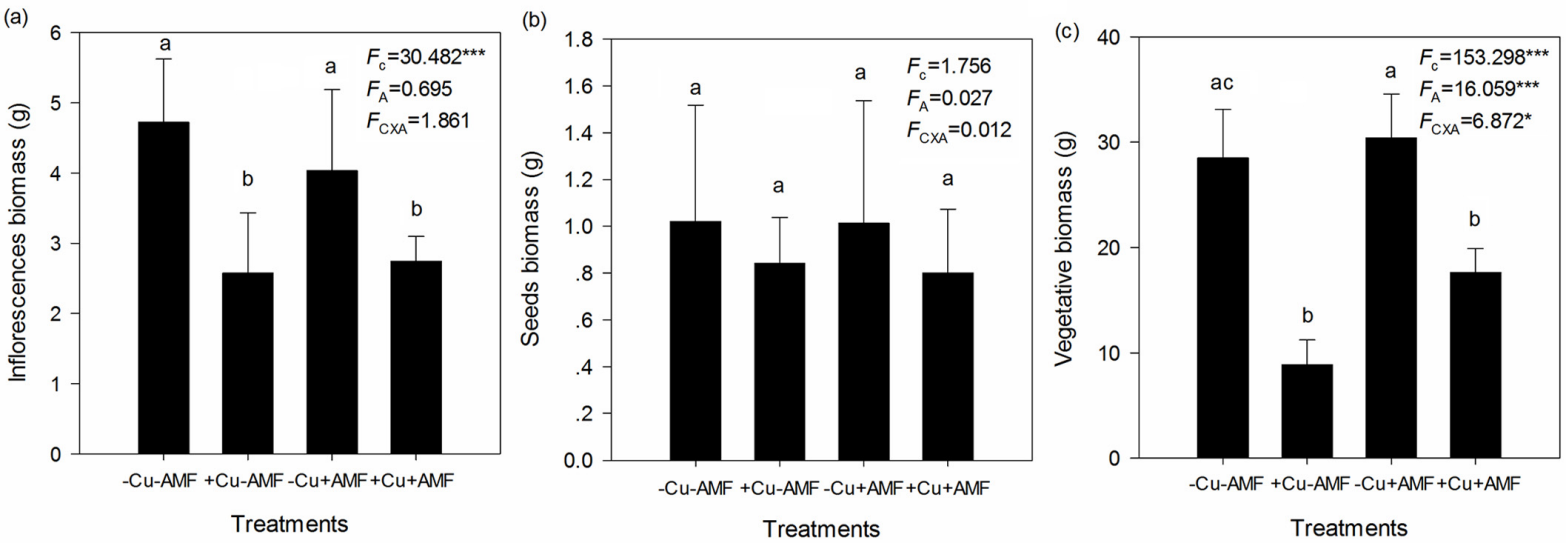
Effects of Cu addition and AMF inoculation on the biomass of flowers (a), seeds (b), and vegetative tissues (c). -Cu-AMF indicates no AMF and no Cu, +Cu-AMF indicates Cu addition, -Cu+AMF indicates AMF inoculation, +Cu+AMF indicates Cu addition and AMF inoculation.

Cu addition significantly decreased inflorescence and vegetative biomass, but had no effect on seed biomass (Fig 4). AMF inoculation significantly increased vegetative biomass, but had no effect on inflorescence or seed biomass (Fig 4). Significant interactive effects between Cu addition and AMF inoculation were found on vegetative biomass, but not on inflorescence or seed biomass (Fig 4).

**Fig 5:**
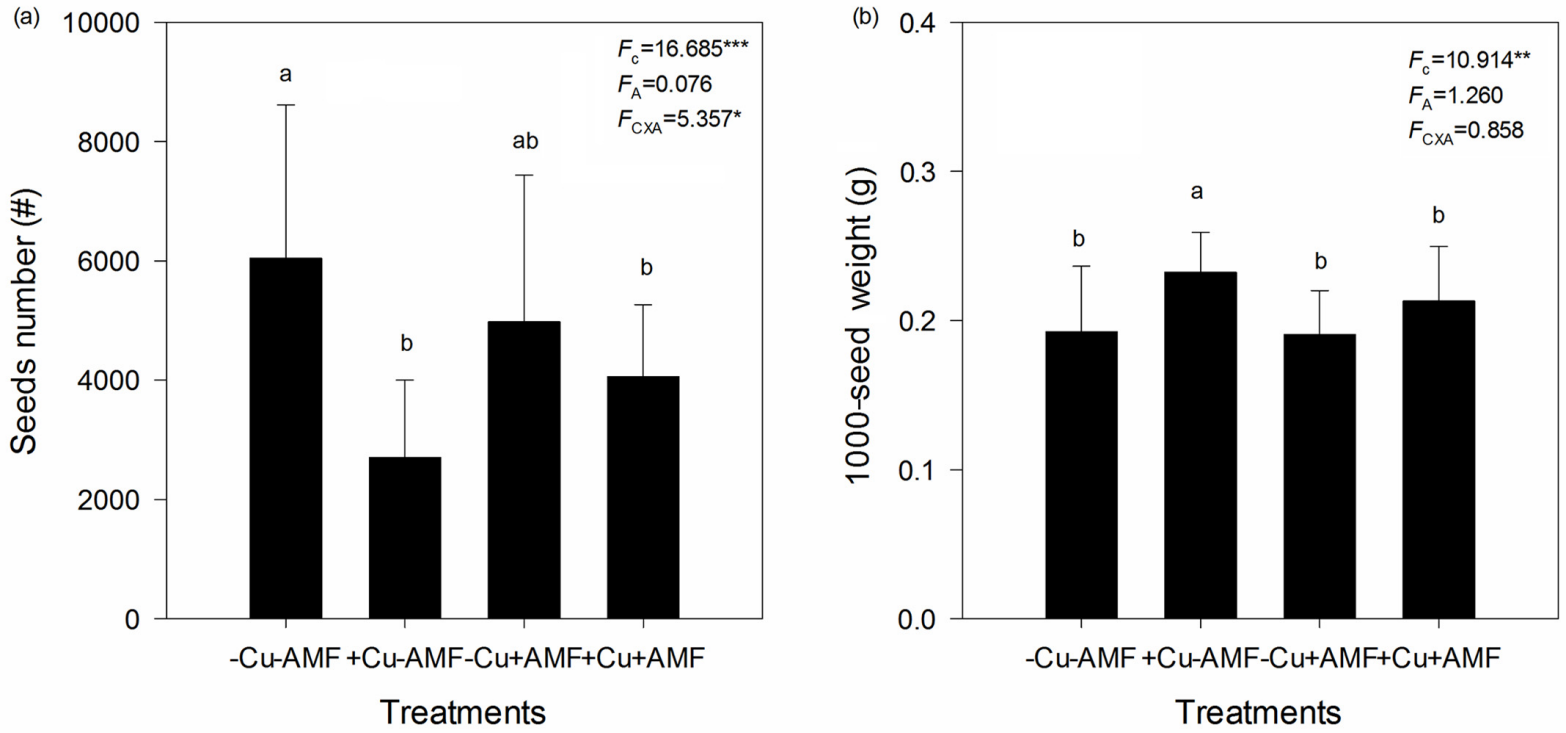
Effects of Cu addition and AMF inoculation on the seeds number (a) and 1000-seed weight (b). -Cu-AMF indicates no AMF and no Cu, +Cu-AMF indicates Cu addition, -Cu+AMF indicates AMF inoculation, +Cu+AMF indicates Cu addition and AMF inoculation.

Cu addition significantly decreased total seed number, but significantly increased 1000-seed weight (Fig 5). AMF inoculation had no effect on total seed number or 1000-seed weight (Fig 5). A significant interactive effect between Cu addition and AMF inoculation was found on total seed number, but not on 1000-seed weight (Fig 5).

## Discussion

Flowering phenology has important demographic consequences for plants [15] and was very sensitive to heavy metals [17]. In this study, Cu addition delayed the onset and the end dates of *E*. *splendens* by up to 18 and 11 days, and also delayed the peak flowering date. The similar effect of Cu addition on flowering phenology was found on *Kummerowia stipulacea* [31]. A longer flowering period could benefit plants with subsequent increases in reproductive output if pollinators are present [15]. In this study, Cu addition decreased the duration of flowering of *E*. *splendens* by 14.4 days, which could result in fewer pollinators and then decreased reproductive outputs.

AMF inoculation has been shown to change flowering phenology [20]. The flowering date of the dominant plant species in limestone soil was advanced after the inoculation of AMF [32]. However, Sohn et al. found that AMF inoculation significantly shortened flowering time of *Chrysanthemum morifolium* compared with non-AMF treatments [33]. In this study, we found that AMF inoculation advanced the peak flowering date of *E*. *splendens* by 6.5 days in the presence of Cu, indicating that AMF inoculation ameliorated the flower date delay and the reduction in flowering duration caused by the addition of Cu. Although no empirical study focused on the interactive effect between Cu addition and AMF, similar alleviation effects of AMF inoculation were reported in a previous study on the negative effects of water stress on inflorescence number of *Biden pilosa* [32]. Ryser and Sauder mentioned that the effects of metal contamination on flowering phenology and reproduction were mostly similar to those caused by water stress, and were not associated with obvious damage to the plants [17]. The recovery of the flowering duration observed here suggests that AMF inoculation could attract more pollinators, thereby increasing the reproductive success of plants and benefit the survival of *E*. *splendens* under Cu stress. In addition, flowering phenology may be related to resource accumulation [20]. AMF infection was positively associated with the increase of phosphorus uptake, which would affect the pattern of flowering [20].

Similar effects of Cu addition and AMF inoculation were found on reproduction of *E*. *splendens*. We found that Cu addition significantly reduced both inflorescence and seed number of *E*. *splendens*, indicating that Cu addition could significantly decrease its reproductive success. Heavy metals are toxic to plants and have significant negative effects on plant reproduction [31]. Negative effects of high Cu concentrations on plant reproduction have been reported previously for many plants species, such as *Hieracium pilosella* [17], *Poa annua*, *Dactylis glomerata*, *Senecio vulgaris*, *Hypochoeris radicata*, and *Andryala integrifolia* [34]. On the other hand, we also found that AMF inoculation significantly increased reproduction, and that there was also a significant interaction effect of Cu addition and AMF inoculation on reproduction. Similar effects of AMF inoculation were also found on the number of flowers of *Petunia hybrida* [19], and on the number of seeds and fruits per plant of *Erodium oxyrrhynchum* and *Plantago minuta* [35]. The alleviating effects of AMF on plants under Cu stress might be due to the reduction in metal uptake or nutritional accumulation in plants induced by AMF infection [9]. The larger inflorescence and seed number of *E*. *splendens* under Cu stress induced by AMF inoculation might have contributed to the survival of plants under Cu stress, as observed for *Rumex dentatus* [18]. These results indicate that AMF infection could contribute to plant tolerance to Cu stress by changing flowering phenology and reproductive success during the adaptive evolution. Further studies are needed to test this hypothesis.

In conclusion, we found that Cu addition significantly delayed flowering phenology, shortened flowering duration and inhibited reproduction of *E*. *splendens*, but AMF inoculation ameliorated the flower date delay and the reduction in flowering duration caused by the addition of Cu. Consequently, AMF inoculation alleviated the negative effect of Cu addition on the reproduction output of *E*. *splendens*. The results provide basic information for developing phytoremediation strategies using *E*. *splendens*.
